# Notes on Surgical Cases

**Published:** 1860-09

**Authors:** E. Andrews

**Affiliations:** Prof. of Surgery in Medical Department Lind University


### NOTES OF SURGICAL CASES.

By E. ANDREWS, M. D.,
Prof. of Surgery in Medical Department Lind University.

*Talipes Varus and Valgus in the same patient. Operation and Cure at twenty-five years of age.*—Mr. L———, of Southern Indiana, aged twenty-five years, called upon me with the view of having a leg amputated, on account of a very bad talipes varus which rendered the limb a burden to him. On examination and inquiry into his case, I found it as follows:

When an infant, the left foot was sound, but he had some disease in the right foot which caused it to turn outward, constituting a case of talipes valgus. As it was never straightened, he grew up walking upon the inner side of the foot, and the end of the malleolus. On examination, I found the member of good shape, but small and illy nourished. The peronei muscles were contracted, and kept the foot in its faulty position. No trace of the tendo achillis could be found, and the muscles of the calf were atrophied. On the whole, it did not seem a difficult thing to rectify the position, though the feebleness of nutrition, and the absence of the tendo achillis gave no hope of a completely perfect limb. The left foot was vigorously nourished, but much more out of position. It was turned inward, in the position of varus, so far, that in walking the sole presented obliquely upward, and the bones were so altered in shape, as to render a complete restoration of the form impossible. The patient stated, that this foot was reduced to its present condition by an accident, which dislocated the ankle when he was a boy, and for some unaccountable reason, the dislocation was not reduced. For this foot he simply desired amputation. After a careful examination, I decided upon attempting a restoration of both feet to the natural position.

Commencing with the left, as being the worst, I severed the tendo achillis and the tendon of the tibialis anticus. Upon endeavoring to straighten the foot, it was found that no amount of force would accomplish it. I therefore made a crucial incision over the external malleolus, and with the saw, resected the ankle joint, removing the lower extremity of the tibia and fibula, and upper part of the astragalus, the whole constituting a wedge shaped mass. The foot was then brought easily around with the plantar surface in its proper position.

On the third day the wound was attacked with erysipelas, which extended to the whole foot, producing several abscesses, and two sloughs. For this, the patient was treated vigorously with tinct. iodine, and ice externally, and mur. tr. iron, every two hours internally. After a few days the erysipelas was subdued, but the ulcerations consequent upon it, were very slow in healing. There was no necrosis. The correct position of the foot was maintained, and long union took place between the astragalus and tibia. At the present time, the last remnants of ulceration are healing slowly, and the foot, though short and *clumpy* in form, will make a very respectable appearance.

Some weeks after the first operation, I proceeded to straighten the right foot, which was affected by valgus. In this case there was much less difficulty. The patient being put as before, under the influence of chloroform and ether. I severed the tendons of the three peronei muscles, and the external lateral ligament of the ankle; then seizing the heal, I forced the member into its proper position. The pressure of the splints and dressings, necessary to maintain the position, was poorly borne by the enfeebled tissues, and several ulcerations occured, nevertheless, the result was a success, and the position of the foot is now correct, and its form perfect.

*Cataract.—Operation by Solution.*—James ———, aged ten years, appeared at Mercy Hospital with cataract of both eyes. As the retina appeared perfect, the patient being able with ease to point out the position of conspicuous objects, I decided to operate. I chose the method by solution. Having dilated the pupil with solution of sulphate of atropine, and anaesthetized the patient, I introduced a cataract needle at the lower part of the cornea, and lascerated the capsule of the crystaline lens. The opaque contents of the capsule began immediately to bulge into the anterior chamber, and the pupil contracted to its former size. On the third day I removed the adhesive straps from the lids, and examined the eye. The contents of the capsule turned still more opaque by the action of the aqueous humour, were projecting in a columnar form into the anterior chamber. There was no inflammation. From this time forward, the case steadily progressed. And at the present time, (six weeks after the operation,) the pupil is nearly clear.

The simplicity and safety of the operation by solution, ought, I think, to commend it to more frequent performance. In the country particularly, where the general practitioner may not have all the instruments, and the experience necessary for a safe performance of extraction, he might still, in many cases, operate by solution; thus restoring the vision, and keeping his patient from being victimized by some wandering quack. Another advantage of this operation is, that it is much safer for the eye, and may be practiced upon both of them without rashness, allowing of course, a sufficient period to intervene between the two operations.

